# Experiences of undergoing internet-delivered cognitive behavioural therapy for climate change-related distress: a qualitative study

**DOI:** 10.1186/s12888-024-06212-1

**Published:** 2024-11-06

**Authors:** Nike Lindhe, Matilda Berg, Kali Andersson, Gerhard Andersson

**Affiliations:** 1 Department of Behavioural Sciences and Learning, Linköping University, Linköping, Sweden; 2 Department of Biomedical and Clinical Sciences, Linköping University, Linköping, Sweden; 3 Department of Clinical Neuroscience, Karolinska Institute, Stockholm, Sweden; 4 Klimatpsykologerna, Stockholm/Malmö, Sweden

**Keywords:** Climate change-related distress, Internet-delivered cognitive behavioural therapy, Thematic analysis, Qualitative, Eco-anxiety

## Abstract

**Background:**

Internet-delivered cognitive behavioural therapy (ICBT) has previously shown promise in effectively treating climate change-related distress. The aim of the present study was to investigate participants’ experiences of undergoing a novel ICBT program targeting psychological symptoms associated with climate change.

**Methods:**

Telephone interviews were conducted with nine participants who had received eight weeks of ICBT for climate change-related distress. A semi-structured interview guide was used to gather information about participants’ experiences of undergoing treatment. Data were transcribed and analyzed using thematic analysis.

**Results:**

The thematic analysis resulted in three overarching themes: (1) Same old feelings, brand new strategies, (2) Bumps in the road, and (3) Personalized climate engagement. Participants experienced that they had developed new coping strategies for handling their emotions, encountered challenges during the treatment period, and adapted their pro-environmental behaviour to their individual needs and circumstances.

**Conclusions:**

The results indicate that participants were able to utilize the treatment material in different ways, resulting in a variety of emotional, cognitive, and behavioural changes. While the treatment was generally described as helpful, participants also raised some concerns regarding the treatment format. These findings can inform further development of ICBT targeting psychological symptoms associated with climate change.

**Supplementary Information:**

The online version contains supplementary material available at 10.1186/s12888-024-06212-1.

## Introduction

Regardless of whether an individual is directly or indirectly impacted, the consequences of climate change have the potential to negatively affect mental health and subjective wellbeing [1]. Varying definitions and terminology have been used in research on emotional responses to the climate crisis, such as solastalgia [2], eco-anxiety [3], and ecological stress [4]. While the conceptual constructs are unclear [5], a broad range of negative emotions have been associated with awareness of environmental threats. For example, fear, sadness, and guilt, including nuances and related forms of these emotions, are commonly discussed in climate emotion research [6]. As climate change poses a severe threat to humanity, anxiety can be considered a rational response, acknowledging the severity of the issue and motivating people to act [7].

However, there are also implications for psychological wellbeing and functioning, as climate anxiety has been linked to depression, stress, insomnia, lower self-rated mental health, and functional impairment [8]. There is some evidence indicating that coping style may influence the effect of negative emotions on the individual. Meaning-focused coping strategies, such as positive reappraisal and drawing on beliefs, values, and goals, have been associated with pro-environmental behaviour and subjective wellbeing [9, 10]. While a scoping review on eco-anxiety interventions identified several papers discussing how mental health practitioners can support clients in developing constructive coping strategies [11], empirical research assessing the effectiveness of such interventions is scarce. Bingley and colleagues [12] presented another systematic review, concluding that although some studies provide qualitative evidence to support the efficacy of climate change anxiety interventions, quantitative evidence is lacking. Further research is therefore needed to establish an empirical basis for interventions targeting this specific problem area [13].

To begin addressing this issue, we conducted a pilot randomized controlled trial (RCT) evaluating the effects of a novel internet-delivered cognitive behavioural therapy (ICBT) program on psychological symptoms associated with climate change [14]. The results were promising, as participants who received an ICBT program called ClimateCope reported lower scores of depression (*d* = 0.87), stress (*d* = 0.76), and climate change-related distress (*d* = 0.79), as well as higher scores of quality of life (*d* = 0.79), compared to a wait-list control group after eight weeks. There were no significant differences between the two groups regarding their climate engagement scores, indicating that although the treatment group increased their wellbeing, they did not significantly differ from the control group in terms of pro-environmental behaviour.

The current study complements the quantitative results from the pilot-RCT by providing a qualitative analysis of participants’ subjective experiences. Undertaking qualitative research can facilitate interpretation of quantitative findings, improve the design and conduct of trials, and enhance effectiveness of future treatments [15]. Qualitative methods have previously been used to investigate various aspects of digital mental health interventions, such as acceptability and usability [16], therapists’ experiences of providing treatment [17], and participants’ experiences of negative effects [18]. Climate change-related distress in psychotherapeutic settings has been qualitatively explored from both patient [19] and therapist perspectives [20]. However, to the best of our knowledge, no published studies have qualitatively investigated digital interventions specifically targeting climate change-related distress.

This study was conducted in order to generate new knowledge and expand our understanding of the recently developed ClimateCope treatment program. More specifically, the aim was to explore participants’ experiences of undergoing ICBT for climate change-related distress and investigate subjective perceptions about the treatment period, the therapeutic material, and outcomes of treatment across various domains using a qualitative approach.

## Methods

### Study design

The study was conducted following a pilot-RCT trial evaluating treatment effects of a novel ICBT program [14]. The study protocol was approved by the Swedish Ethical Review Authority (dnr 2021–0523). A semi-structured interview guide was developed and used to collect qualitative data on the participants’ personal experiences of undergoing treatment for climate change-related distress. Data were analyzed using inductive thematic analysis [21].

### Participants and recruitment

The original sample in the pilot-RCT consisted of 60 participants who were recruited primarily via advertisements on social media platforms. Potential participants registered by signing an informed consent sheet and completing a set of questionnaires on the study webpage. Telephone interviews were conducted to ensure that each potential participant met all inclusion criteria while meeting none of the exclusion criteria. Further information about the recruitment process is presented in the original report [14].

For this qualitative study, a sub-set of the original sample was used. All 60 participants were sent a questionnaire one year after finishing treatment, including self-report measures and a question about whether or not they were willing to take part in an optional telephone interview. Out of the 23 participants who completed the questionnaire, nine participants (39%) opted in, and were subsequently included in the current study. Thus, a sample consisting of nine participants who had previously undergone treatment for climate change-related distress was obtained.

All nine telephone interviews were conducted between June and August 2023. The mean age of the interviewed participants was 39 years (range 26–56). Eight of the participants were women and one was a man. Four participants had previously been in the treatment group and five in the original wait-list group before receiving treatment. All participants except one had completed college or university. All participants were employed at the onset of treatment, although three participants were not actively working due to parental or sick leave. The self-report questionnaire ClimateCope Index (CCI-15) was used to measure participants’ climate change distress, and all individuals scored lower on CCI-15 post-treatment compared to pre-treatment. An overview of participant characteristics is provided in Table 1.

**Table 1:** Participant characteristics

Participant	Age	Gender	Highest Education Level	Employment Status	CCI-15 Change Score
1	30–34	Female	College/university	Employed	– 31
2	35–39	Female	College/university	Employed	–20
3	30–34	Female	College/university	Parental leave	–8
4	25–29	Female	College/university	Employed	–21*
5	30–34	Female	College/university	Registered sick	–5
6	50–54	Female	College/university	Employed	–21
7	55–59	Female	College/university	Employed	–11
8	25–29	Female	College/university	Parental leave	–15
9	50–54	Male	Secondary school	Employed	–12

* As post-treatment data were missing for participant 4,* this is the CCI-15 change score between pre-treatment and 1-year follow-up*.

### The ClimateCope treatment program

The treatment was an 8-week ICBT program with the aim to reduce symptoms of psychological distress related to the ongoing climate crisis. It consisted of 15 modules, each with a specific focal point (for a more detailed overview, see 14). All participants received the “Introduction” module and the “Conclusion and maintenance plan” module. For the remaining six weeks, a selection of modules was made based on the participant’s self-report measures, the clinical telephone interview, and the participant’s requests. All modules were built upon the same structure, including psychoeducation, exercises, and worksheets. Due to the nature of the emotions being targeted, the material was explicit in not questioning the reality or magnitude of the ongoing climate crisis. An additional focus was directed towards validating participants’ negative responses while aiming to enhance appropriate coping strategies and encouraging each participant to act in line with their values.

All questionnaires and modules were administered via the Iterapi platform [22]. Here, participants also received weekly feedback via text message from their assigned therapist for the entirety of the treatment period. In cases where additional support was needed, participants had the option to send a text message to their therapist at any time and receive an answer within the next 24 h. The group of therapists consisted of five MSc psychology students who had completed their clinical training. All therapists were supervised by three licensed clinical psychologists, experienced in providing ICBT. Furthermore, all therapists had the possibility to consult a psychiatrist if a need for medical expertise emerged.

### Procedure

A semi-structured interview guide was developed to collect qualitative data about participants’ personal experiences of undergoing treatment for climate change-related distress. The first author formulated open-ended questions, drawing inspiration from earlier qualitative work on transdiagnostic ICBT [23, 24]. The fourth author reviewed the interview guide which, in its final version, comprised seven questions related to three different topics: participants’ views on the treatment, their experiences of eco-anxiety, and their own engagement in climate change-mitigating behaviours. When deemed appropriate, probes and follow-up questions were added to encourage participants to elaborate their responses. To stay consistent with the terminology used in the treatment material, the term “eco-anxiety” (in Swedish, “*klimatångest”*) was used in the interview guide. In order to avoid restricting the participants’ accounts of their emotions, the term was described to the participants as including a wide range of emotions, such as worry, sadness, guilt, and frustration, for example. A full copy of the interview guide is available in Supplementary Material 1.

All nine telephone interviews were conducted by the first author who was also involved as a co-creator of the treatment material. She served as one of the therapists during the treatment period, and had been in previous contact with several of the participants via text message and/or telephone. Consequently, the first author had a good understanding of the treatment program’s contents prior to performing the interviews. A digital audio-recorder was used to enable verbatim transcription, and the length of the recordings ranged between 14 and 23 min. Information about the study was repeated before the recording started, and participants were reminded that their anonymity would be maintained throughout the research process, as well as in the write-up of the study. The final transcripts were not shared with the participants for further comments.

### Data analysis

Data were analyzed following Braun and Clarke’s [21] guidelines for conducting thematic analysis (TA). TA is a flexible, accessible method that can be used to generate detailed descriptions and rich interpretations of subjective experiences and perceptions. A common approach within the TA research community is embracing researcher subjectivity as a resource, defining meaning not as being fixed within data, but rather as contextually situated, partial, and provisional [25]. During the analytic process, a bottom-up, inductive approach was used. Inductive TA was chosen since it aligned well both with the study’s exploratory aim and the nature of data, which were retrieved from relatively brief telephone interviews. This qualitative method also allowed codes and themes to be driven by data rather than constrained by predefined categories, which was considered beneficial to the analytic process.

During the first stage of the analysis, the first author made herself familiar with the data by listening to the audio files of the interviews, transcribing them verbatim, reading, and re-reading the transcripts. Coding was conducted semantically, aiming to capture explicit statements relevant to the research aims. Preliminary themes and subthemes were first generated, then reviewed in order to ensure a good fit between these broader categories and the coded data extracts. Subthemes that were not sufficiently supported by participants’ quotes were removed. Finally, themes were named and refined in collaboration with the second author, who had prior experience conducting thematic analysis. All decisions were made through discussion until consensus was reached. The results of the analysis were reviewed and approved by the third and fourth authors.

### Reflexivity

As the analysis was conducted using subjective interpretation, it should be situated and understood within its context. The first author was involved in developing the treatment program, served as a therapist during the treatment period, and conducted all interviews, which is likely to have affected the way that participants’ statements were interpreted. Potentially, the first author’s preconceptions of the treatment and participants contributed to a less inductive, bottom-up approach to the collected data. However, this prior knowledge and experience may also have facilitated the analytic process, allowing the first author to identify relevant themes and nuances within the data.

The second, third and fourth authors were also involved throughout the project and supervised all therapists during the treatment. The second and fourth authors both had prior experience in conducting thematic analysis and had authored several publications using this qualitative method. The first author had theoretical knowledge of the analytic method from coursework and engaging with relevant literature, but had not previously applied it practically in a research context. The analysis, discussion, and conclusions are results from collaborative work between all four authors, given their professional experiences, subjective understandings, and the context within which the project was carried through.

## Results

During the analysis process, three main themes were identified: (1) *Same old feelings*,* brand new strategies*, (2) *Bumps in the road*, and (3) *Personalized climate engagement*. Aiming to describe a multitude of facets, these themes were further divided into a total of nine subthemes, here presented with numbers corresponding to the three main themes: (1) *Useless suffering*,* Sensible worrying*,* Learning to climate cope*, (2) *Lacking time and energy*,* Life gets in the way*,* Longing for connection*, and (3) *Taking more action*,* Finding a balance*,* Sticking to what you know.* In Table 2, an overview of all themes and subthemes is available. The quotes presented in the following sections were selected with the intention to provide a rich and illustrative view of the contents of each theme.

**Table 2:** Overview of themes and subthemes

Theme	Subthemes
1. Same old feelings, brand new strategies	1.1 Useless suffering 1.2 Sensible worrying 1.3 Learning to climate cope
2. Bumps in the road	2.1 Lacking time and energy 2.2 Life gets in the way 2.3 Longing for connection
3. Personalized climate engagement	3.1 Taking more action 3.2 Finding a balance 3.3 Sticking to what you know

### Same old feelings, brand new strategies

When reflecting on their climate emotions, many participants concluded that the way they felt about climate change had not fundamentally changed. However, they described a shift in how they perceived and dealt with their emotions. Instead of becoming overwhelmed by worry, anxiety, and negative thought spirals, they had improved their ability to take a step back and observe their inner experiences with greater awareness. Thus, participants expressed a sense of control over their negative responses to the climate crisis. This main theme was sorted into three subthemes, namely *Useless suffering*,* Sensible worrying*,* and Learning to climate cope.*

#### Useless suffering

While undergoing treatment, participants started to reconsider the usefulness of their negative responses to the climate crisis. By taking a new perspective on the effects of their own suffering, they realized that the fact that their daily lives were being severely impacted did not yield any positive results; neither for themselves, nor the climate. One participant put it into words in this way:

*I know that even if I feel worse, it doesn’t mean the climate gets better in any way. So, the best thing is if I feel as good as possible, that’s one way to think about it. – Participant 5*

In this case, the participant concluded that suffering did not impact the climate in a helpful way. As “feeling worse” was deemed inequivalent to “the climate getting better”, it rendered itself useless. Another participant used a metaphor of air travel to describe a similar experience:

*“Speaking of flights*,* when they do the safety briefing*,* you actually have to put on your own oxygen mask before you can help anyone else. I can’t help anyone else if I myself am falling apart.”* – *Participant 7*

The sentiment conveyed here was that in order to be able to actively engage in climate change mitigation behaviour, the participant had to make sure that she herself was strong enough to do so. This may be understood as a heightened awareness of the importance of prioritizing and attending to her individual needs in order to be able to adequately care for the needs of others. For this participant, suffering was no longer seen as an unpleasant mean to an end, but rather as an obstacle standing in the way of reaching her desired goals in a sustainable way.

As illustrated by these two excerpts, participants described gaining new perspectives on their emotions, where severe distress was no longer seen as directly associated with benefitting the climate. With the insight that they might be able to simultaneously feel good and do good, efforts to reduce their own suffering were seen as more justified.

#### Sensible worrying

In addition to reappraising suffering in relation to the climate crisis, participants also described shifts in perspective regarding their own worrying. While actual suffering was regarded unnecessary for the cause, worrying was rather seen as largely sensible. Manageable levels of worry were described by one participant as reasonable and even helpful:

*I think that people should be worried about the future and the climate. Everyone should have a certain level of concern; I feel like it would be healthy because of the way things are. Maybe not to the point where you almost get palpitations just reading about it, or just feel sad and powerless, but so that you can try to turn it into an act of power. – Participant 6*

This participant highlighted the inherent rationality of experiencing concerns about climate change. However, being worried was simultaneously depicted as a balance act; too much of it can make you feel powerless, but just enough worry may constitute a useful tool. This fine balance was illustrated by another participant, who was still experiencing worry, but in a different way than before:

*“It’s more like a sober concern*,* it’s like*,* seeing it more clearly and seeing it for what it is. I don’t feel this sort of doomsday panic like I used to. I am worried and I’m not naïve*,* I know what’s going on and what the risks are. But it doesn’t really affect me in my daily life*,* even though I often think about it during the day. Not at all in the limiting way as before.”– Participant 1*

No longer in a state of “doomsday panic”, this participant regained her ability to disentangle rational concern from more irrational catastrophizing. Furthermore, she became more able to maintain a sensible level of worry without allowing excessive worry to spill over into her daily life and limit her functioning.

This subtheme captured some of the complexity associated with participants’ negative responses to the climate crisis. While some degree of worrying was still considered sensible and functional by participants after undergoing treatment, a mind entirely riddled by worry was rather seen as a potential threat to their wellbeing and ability to take action.

#### Learning to climate cope

Alongside experiencing shifts in perspective regarding suffering and worry, participants described developing various new strategies to cope with their negative emotional and cognitive reactions. When asked whether she found any aspects of the treatment helpful, one participant mentioned that she appreciated the presented general approach to managing emotions. She had started practicing applying this approach on her own, describing it as follows:

*Kind of… Accepting that ‘this is how I feel right now, but it won’t last forever’ and then – finding the courage to embrace that emotion. – Participant 3*

Her new coping strategies included acceptance and engagement, in contrast to previous patterns of resistance and avoidance. While acknowledging the volatile nature of her emotions, she now strived towards embracing and fully experiencing them. Another participant gave an example of how she utilized the treatment’s cognitive exercises in order to manage worrisome thoughts:

*“Before*,* I probably thought about it very*,* very often*,* almost daily*,* but in a negative way. Maybe I still think about it every day*,* but more like – well*,* it doesn’t always have to be so negative. It could be something like*,* now this law has passed or just trying to look at some positive news as well.”* –* Participant 8*

This participant still thought about climate change daily, but had developed a more flexible and balanced mindset by actively taking note of messages that stimulate hope. A similar strategy was mentioned by another participant:

*“My worry for the future is probably still there*,* but the daily anxiety might be a bit less*,* and perhaps more towards trying to… Well*,* I try to focus on what I can do and my actions and choices.”* –* Participant 4*

By redirecting her focus to what she could actually do about the issue, this participant had initiated a process of channeling unpleasant emotions and thoughts into constructive action. What is noteworthy here is that participants did not seemingly attempt to escape or suppress their negative thoughts and emotions as a result of undergoing treatment; instead, they described learning alternative strategies for climate coping that felt suited to their individual needs. Thus, “old” feelings could persist, but be handled in a new, more constructive manner.

### Bumps in the road

Although many participants expressed appreciation for the treatment program and noted positive effects during and after the treatment period, they also reported facing challenges along the way. Time constraints, conflicting commitments, and frustration with the digital format were some obstacles that were mentioned by participants. These are further elaborated on in the following three subthemes; *Lacking time and energy*, *Life gets in the way*, and *Longing for connection*.

#### Lacking time and energy

Several participants mentioned that the treatment period was demanding in terms of the effort required to complete the modules. Some struggled to keep up with the pace of the treatment, as new modules were assigned every week:

*“There were short intervals in between for me to catch up and properly settle in everything*,* along with everything else. I might have needed a bit more time in between to really be able to do it the way I wanted.”* – *Participant 2*

For this participant, the primary issue seemed to be that the fixed deadline for treatment termination hindered her from exploring the treatment material more in-depth. Consecutively working through one module after another was seen as challenging, and her suggestion for improvement was to lengthen the duration of the treatment period. Furthermore, some participants described lacking the energy needed to deal with emotions that were evoked by the treatment content:

I thought it was a bit challenging, having to address these, like, monsters that you had tucked away quite carefully, and then look at them and talk to them. – *Participant 1*

This quote captures the difficulty of facing issues that had been avoided for a long time. Processing unpleasant emotions and assimilating new experiences demanded both time and mental resources from this participant. This subtheme reflects the strain that some participants felt was imposed on them by the treatment’s format and content. A common perception among participants seemed to be that receiving more time would have relieved some stress, enabling them to engage with the material on their own terms.

#### Life gets in the way

In addition to the treatment in itself being time- and energy-consuming, participants found that various aspects of their daily lives interfered with their ability to work with the modules. As other tasks and commitments called for their attention, participants found themselves prioritizing more pressing needs over spending time on texts and exercises on the treatment platform.

*“Unfortunately, it coincided with the fact that I had so terribly much to do at work and with other commitments, so it felt like I couldn’t really dig into it with the full focus that I would have wanted.” – Participant 7*

Despite this participant’s previous ambition to wholeheartedly focus on the treatment, commitments in and outside of work interfered. This led her to engage less in reading and doing exercises than initially intended, which seemed to become a source of frustration.

In addition to managing daily chores and keeping up with their schedules, participants also had to cope with external circumstances beyond their control. For example, the treatment period coincided with the Russian invasion of Ukraine in February 2022, which caused much concern among the participants.

*The climate is obviously very important, but there might be other things to worry about that kind of take over, so it fades into the background. You might think that it is… Well, not exactly trivial, but it takes a step back at least. It’s like, preventing a world war might feel more important at that particular moment. – Participant 6*

One implication that can be drawn from this quote is that the participant’s level of worry about the escalating conflict was so high that it, at least partly, overshadowed many other foci of worry. Although still caring deeply for the climate, another urgent global issue took precedence in her mind, reducing her motivation to adhere to treatment.

What these two quotes collectively illustrate, although on rather different scales, is the challenge of finding enough resources, both practical and cognitive, to incorporate the treatment into everyday life. Despite feeling motivated to engage in treatment by the time of registration for the study, unforeseen events occurred that hindered some participants from fully doing so.

#### Longing for connection

As the treatment was administered via a digital platform [22], participants communicated with their therapists solely through written text messages. This was described by some participants as contributing to a sense of distance, and not fulfilling their desire to actually talk to someone about their struggle. One of the participants reflected on the ICBT format and how well it aligned with her personal needs and preferences:

*“I know that many of the exercises were about connecting to others, but there was still a pretty big focus on the individual; it’s kind of inherent in the nature of CBT. My experience is more about needing a space to talk, so it probably would have been more helpful if it had been more like, well, a conversation with a psychologist.” – Participant 3*

Working individually on creating social connections was not seen as sufficient for this participant, who would have preferred being able to talk to her therapist during the treatment period. Although she had a prior understanding that the treatment was meant to be focused on the individual, she appeared somewhat disappointed with how it had played out in practice.

Another participant expressed a similar thought:

*“I feel like it’s better to meet a person, to be two. Of course, that’s a different thing.” – Participant 9.*

Here, the concept of two people being together in a therapeutic setting was compared with undergoing therapy through a computer screen. For this participant, the former option would have been preferable, as it would allow for a deeper connection with the therapist. This subtheme highlighted the need for some participants to share difficult emotions and thoughts related to climate change in a more socially dynamic manner. While some participants did not mention any challenges with the digital format, others explicitly expressed that this was a barrier in their therapeutic process.

### Personalized climate engagement

Climate engagement was perceived as important by all participants, but the treatment influenced their approaches to taking action in different ways. While some participants increased their commitment, others maintained the same level of engagement or shifted their focus to increase their own wellbeing. Despite taking different courses of action, the treatment helped participants reflect on their individual positions within the climate movement and consider how to tailor their climate engagement to their personal situations. Three subthemes were generated in order to capture the diversity within the main theme: *Taking more action*,* Finding a balance*, and *Sticking to what you know.*

#### Taking more action

Although the primary aim of the ClimateCope program was not to increase participants’ pro-environmental behaviours, some participants extended their efforts in climate change mitigation. One of the participants reported that undergoing treatment had motivated her to, in her own words, “practice what she preached” to a larger extent. She illustrated this with an example:

*Changing the heating system is very expensive, but I actually prioritized switching to an environmentally friendly one. And it just feels really good – to act on what you feel. – Participant 2*

Here, she had chosen to take individual action by replacing her old heating system with a more energy-efficient alternative, which would provide environmental benefits in the long-term. Her decision aligned well with her personal values, bringing her a strong sense of contentment. In addition to individual efforts, several participants also highlighted the importance of engaging in collective action:

*“I’ve had an intention to engage in climate activism but haven’t had the energy, courage, you know. But when I got some distance from it and could formulate that it was important to me, then I managed to take that step. I was a bit stuck before, so it helped me break free.”– Participant 1*

By reflecting on personal values and rationally contemplating her situation, she gained the internal strength she needed to push herself over the edge of her comfort zone. She described her former self as “stuck”, as if she had previously been trapped in a passive state. Through the process of undergoing treatment, she found herself able to “break free” and pursue her convictions. Another participant expressed that she had increased her awareness of the importance of public engagement:

*“Maybe I’m a bit more aware of putting pressure towards the top, towards politicians and being more active there. I might have been active before in my life and in daily routines, but… Not just sitting and thinking about it alone but also being involved and seeing what’s happening in organizations working for the environment and in politics. Perhaps a bit more than I did before.”– Participant 4*

While she had engaged in individual pro-environmental behaviour for a long time, she had now realized that she could make a more significant impact by joining a group of people working for the same cause. The quotes suggest that the participants did not necessarily change their previous positions in relation to climate engagement. Rather, they seemed to have come closer to their personal values by evaluating their situations and considering new options for taking action.

#### Finding a balance

While undergoing treatment, some participants recognized an imbalance between their focus on climate change mitigation and their attention to personal wellbeing. In their efforts to reduce their carbon footprint as much as possible, they seemed to have stumbled into the pitfall of eco-perfectionism, scrutinizing their own consumption patterns and lifestyle choices. For these participants, finding a sustainable, long-term balance between active commitment and recovery involved changing or even reducing their pro-environmental behaviour. One participant described her experience in this way:

*“There are so many areas, like home purchases and transportation, and, yeah, there are so many areas where you feel like you have to do a lot. So, I try to remind myself that sometimes I just have to take a break and think that, you know, I need to gather energy to tackle things again later.” – Participant 8*

This quote illustrates the emotional toll experienced by the participant while trying to make conscientious and ethically sound decisions regarding a large number of complex issues. While she was still motivated to engage in pro-environmental behaviour, she had come to the realization that it was okay, and even necessary for her, to take a break from time to time to focus on feeling better herself. Another participant had reached a similar conclusion:

*“It has probably become somewhat less, because I’ve tried to sort things out, because I had so much else going on. I’ve sold one of my companies, I’ve turned down some volunteer assignments, and this also affects my climate engagement a bit. I actually have to choose to opt out of some things for a while to keep my commitment at a reasonable level.” – Participant 7*

She described how she had initiated a process of scaling down and removing some elements out of her tight time schedule. The decision to do so was described without notable emotional expression, as if she had pragmatically considered her options and concluded that this was the most suitable approach.

This subtheme brings forward participants’ reflections on striving for sustainability not only environmentally, but also in their personal lives. While still engaging in pro-environmental behaviour, they had recognized the need to prioritize and make short-term adjustments to obtain a more balanced and sustainable long-term engagement.

#### Sticking to what you know

Rather than aspiring to become more engaged or to put an additional focus on personal wellbeing, some participants seemed most comfortable with maintaining the same level of commitment. For these individuals, a personalized climate engagement meant continuing on the same path as before undergoing treatment.

*“I just have a very strong commitment to a lot of things. I’ve had a significant awareness of environmental and climate issues for over 30 years, at least 35 years now. It’s something that has been present for as long as I can remember, actually.” – Participant 9.*

With a long background of engaging in the climate movement, this participant did not feel the need to change what he perceived as a functional approach. His commitment seemed to be closely interwoven with other aspects of his being, having developed over decades, and ultimately turned into a lifestyle. Another participant shared that she felt that her medical condition affected her ability to participate in certain types of activities:

*“It’s like a balancing act in terms of how much, well, how many new people you should meet and such. But, well, it hasn’t diminished at least, so it’s about the same.” – Participant 5.*

While she had considered taking more collective action, she was also keen not to exacerbate somatic symptoms by overstraining herself in, for example, social situations. Thus, she had chosen to adhere to the pro-environmental behaviours that she knew were feasible.

Contrasting the other two subthemes, this third and final one focused on participants’ experiences of being content with their existing climate engagement. Rather than adopting behavioural changes as a result of treatment, they seemed to have opted for adhering to habits that already were aligned with their individual aspirations and needs.

## Discussion

The three main themes and nine subthemes identified in this study cover a broad range of experiences associated with undergoing treatment for climate change-related distress. A variety of emotional, cognitive, and behavioural changes were described by participants, as well as challenges during the treatment period. While the treatment was perceived as helpful in general, it became apparent that participants utilized the material in different ways depending on their unique needs and circumstances. With the development and evaluation of the ClimateCope treatment program being the first of its kind, these are novel findings that may contribute to our understanding of how interventions targeting psychological symptoms associated with climate change can be perceived and utilized by individuals.

The first theme, “Same old feelings, brand new strategies”, captured participants’ experiences of gaining new perspectives on their negative responses to climate change, alongside developing constructive coping strategies. New insights and positive behaviour changes appear to be two recurring themes in qualitative analyses of interview data from participants who had undergone ICBT for various transdiagnostic issues. Similar results have been found in a study of ICBT for low self-esteem [23], where participants reported reaching a widened understanding of themselves and their emotional experiences. Moreover, participants’ experiences of acquiring practical skills and helpful strategies were observed in a study of ICBT for perfectionism [26]. Thus, the presented findings add to an expanding body of research indicating that ICBT has the potential to bring about positive changes that are not only evident in scores on self-report measures, but also in verbal accounts of participants’ subjective experiences.

Despite sharing similarities in format and findings, a notable difference between ClimateCope and other transdiagnostic ICBT treatment programs is in the nature of the underlying source of symptoms. Given that the climate crisis is a real, tangible threat that cannot be eliminated by the efforts of any single individual, participants have limited control over the root cause of their problems. Thus, ClimateCope puts more emphasis on external concerns by addressing macro worries while promoting meaning-focused coping on the micro-level. Another example of this approach can be found in an evaluation of ICBT addressing psychological symptoms related to the COVID-19 pandemic [27], where the focus was on individual change within a context of significant societal challenges.

In the present study, the additional emphasis on meaningfulness can be discerned among participants’ experiences of developing new strategies to deal with negative responses. For example, participants mentioned actively seeking positive climate news as a way to view their current situation in a less negative light. As discussed by Park and Folkman [28], cognitive reappraisal strategies, such as finding small victories and focusing on events that reinforce existing goals, may facilitate keeping things in perspective and maintaining a sense of meaning under chronically stressful conditions. Shifting focus towards feasible mitigation behaviours and framing worry as a source of empowerment are additional examples of how participants utilized positive reappraisal and found meaning while still acknowledging the stressor.

As meaning-focused strategies can buffer negative affect and help individuals constructively cope in a difficult situation [29], participant’s accounts of developing such are noteworthy in light of the quantitative findings from the pilot-RCT which indicated that wellbeing increased as a result of treatment. There is some research that has identified coping style as a mediator between stressful life events and mental health outcomes [30, 31]. Whether meaning-focused coping plays a significant role in decreasing climate change-related distress is a subject for future inquiry. Regardless, the first theme in the analysis indicates that meaning-focused strategies can be helpful when dealing with a chronic macro stressor on the micro level.

The second theme, “Bumps in the road”, highlighted various challenges encountered by participants during the treatment period. While most participants described their overall experience as positive, they also noted some negative effects, such as the intervention being demanding in terms of effort. One of the most frequently brought up issues was not having enough time, energy, and capacity to work through the treatment material at the intended pace while managing other things in an already busy schedule. As new modules were assigned every week, participants felt that they were pressed for time and unable to fully assimilate all components of the intervention. One participant proposed that longer intervals between modules would have made it easier for her to fully settle into the material. A similar request was highlighted in a qualitative evaluation of ICBT for informal caregivers [32], where participants wished for more time to reflect on emotional and cognitive responses elicited by the intervention. Lack of time has been identified as a theme in other qualitative analyses of ICBT trials, described by participants as a factor hindering engagement [33] and increasing stress levels [18].

As the ClimateCope intervention was perceived as both time-consuming and emotionally demanding, it is possible that a wider time frame would have alleviated some of the pressure on the participants. Alternatively, presenting information in a more concise way might have led to less time reading, thus allowing participants to focus on processing new information and conducting exercises. Preliminary results from a study conducted by Karlsson-Good and colleagues [34] suggested that condensed, shorter-text versions of ICBT may be equally effective as full-text versions, while having the additional advantage of reducing effort for participants. Thus, making adjustments to the treatment material might be a strategy to consider for reducing stress in participants. On the other hand, there is a risk if treatments are too brief as such treatments also may result in dropout and smaller effects.

Another challenge brought up by participants was the fact that therapist-contact was administered exclusively via text-message during the treatment period. Some previous qualitative evaluations of digital interventions offering online support (e.g., [35, 36]), have captured participants’ desires for having face-to-face-contact with their therapist. Meanwhile, there seems to be individual differences among participants’ preferences for type of support. As noted by Bendelin and colleagues [37], having face-to-face-contact may be perceived as more or less important depending on participant characteristics. Results from a systematic review and meta-analysis suggest that therapist-supported ICBT yields similar effects as face-to-face CBT for both psychiatric and somatic disorders [38]. However, the authors recognized that there are still uncertainties regarding moderators of these treatment effects, and propose that face-to-face CBT might be a better fit for some individuals. Thus, the possibility remains open that some of the participants in the present study might have had different outcomes and subjective experiences from undergoing treatment had they been provided with the opportunity to communicate with a therapist in a non-digital setting. In future trials, offering brief telephone calls in addition to text messaging could be a way to meet participants’ requests for better communication with their therapist.

The third theme, “Personalized climate engagement”, offered insight into how participants perceived their own mitigation actions. All participants shared reflections on what they believed would be feasible for them in terms of personal engagement, given their external circumstances and individual resources. In this sub-sample, pre-treatment scores on pro-environmental behaviour were generally high, yet some participants further extended their efforts in climate change mitigation following treatment. This can be seen as a positive outcome, as it shows the treatment’s potential to address environmental needs as well as individual needs. One participant in particular underscored the significance of elucidating personal values, claiming that this was what helped her take the next step towards engaging in climate activism. The concept of personal values has been explored in much of the literature on climate change mitigation, for instance, as part of Stern’s [39] value-belief-norm-theory that has been found to effectively predict pro-environmental behaviour [40, 41]. Furthermore, strong biospheric values seem to be positively associated with taking climate action [42, 43]. As the ClimateCope intervention included multiple exercises focusing on values and goals, it is possible that these became more salient for participants during the course of treatment, thereby increasing participants’ motivation to engage in climate change-mitigating behaviours.

After undergoing treatment, some participants concluded that decreasing their pro-environmental behaviour to some degree was necessary for their wellbeing. Given that human behaviour and lifestyle changes are needed to reduce greenhouse gas emissions [44], it could be argued that participants reducing their climate commitment is an undesirable outcome. Bingley and colleagues [12] particularly highlighted the importance of thoroughly evaluating interventions, so as to avoid unintentional effects on, for example, societal or environmental scales. However, the presented findings should be interpreted in light of the characteristics of the recruited sample. Since it mainly consisted of individuals already deeply involved in climate change mitigation, it is understandable that some felt the need to set aside part of their commitment to focus on improving their own wellbeing during the treatment period.

While taking collective action has been found to increase constructive hope [45] and provide social support [46], it also has the potential to exacerbate psychological symptoms, particularly in cases where there is a lack of adequate self-care strategies [47]. Clayton [48] has suggested that climate engagement may be more or less beneficial to the individual depending on their level of distress. When participants reflected on their personal situations, they emphasized the importance of finding a manageable level of commitment to be able to efficiently address challenges. Thus, participants expressed desires to keep engaging in mitigating behaviour, although in a way that would be feasible for themselves. Similar experiences have been portrayed in a study by Howard [49], where participants occasionally withdrew from activist spaces as a strategy to emotionally recharge. In the present study, participants’ accounts of reducing their pro-environmental behaviour are interpreted as examples of how highly engaged individuals may strive to find a long-term sustainability within their engagement. However, treatment effects on both individual and environmental outcomes should be carefully evaluated in future studies to reduce the risk of undesired consequences, such as participants avoiding discomfort associated with climate change by adopting a passive stance.

The qualitative analysis discussed here complements the quantitative findings [14], showing that ICBT can be a useful tool in addressing climate change-related distress. Participants’ reflections on undergoing treatment offer insight into how individuals may navigate and make sense of their experiences while trying to cope under the presence of a threat as imminent as the climate crisis. Additionally, participants’ feedback contains valuable information on how the treatment program could be refined and adjusted in order to provide adequate support for those in need. Building on the presented findings and further exploring the potential of ICBT in the climate context is an objective for future research. Given that the evidence on effective interventions targeting climate change-related distress is limited [12], and with the consequences of climate change becoming increasingly severe [44], this area of research is highly relevant.

## Limitations

The presented findings contribute to a greater understanding of participants’ experiences of undergoing treatment for climate change-related distress. However, conclusions should be drawn with some consideration, as there are several limitations to this study.

First, the number of interviews was small, as only nine individuals agreed to participate in a follow-up interview. This potentially constrained the diversity and range of perspectives within the data set, rendering themes and subthemes that may not be representative for all participants that received the ClimateCope treatment. However, the aim when using thematic analysis as a method is not typically to achieve “saturation”, where additional themes or codes are no longer yielded [50]. Furthermore, attempting to generalize results from a representative sample to a larger population is a practice that is more closely related to quantitative research methods [51]. Thus, the perspectives shared by these nine participants hold significance on their own, regardless of whether a larger sample size would have resulted in additional or different themes.

Second, interviews were conducted approximately one year after the treatment ended. It is likely that this affected the participants’ ability to accurately recall their experiences during the treatment period. However, the memories retained by participants and the ways in which change processes have had time to proceed over the course of one year are of interest and bear meaning in their own right. If the analysis had taken place right after the treatment period, participants would most likely have made other statements regarding, for example, their engagement, as eight weeks of treatment may be insufficient time to make significant behaviour changes, such as joining groups or attending public demonstrations.

Third, it is difficult to draw conclusions about the extent to which the participants’ experiences of positive change are attributable to the intervention. In some cases, participants were able to provide clear examples of how they had utilized the modules’ exercises and information. In other cases, they offered more vague descriptions of how they had benefitted from the treatment, such as perspective shifts that seemed to have evolved gradually over a longer period of time. As various external factors may have affected the participants’ wellbeing and behaviour during and after treatment, uncertainty regarding causality still remains. In this study, however, we have aimed to investigate participants’ experiences of undergoing treatment, focusing on what they themselves have found to be helpful, rather than on objective mechanisms of change within the treatment process.

Finally, the participants’ descriptions of their experiences may have been influenced by biases introduced by the recruitment method and the interview setting. Conducting interviews as late as one year after treatment termination potentially reduced the response rate and contributed to sampling bias, as participants with positive experiences may have been more inclined to agree to a telephone interview. In this sample, all nine participants reported lower scores of climate change-related distress following treatment, meaning that those who did not experience improvement were not represented. Furthermore, the first author’s prior involvement in the treatment may have contributed to a social desirability bias. It is possible that participants provided mainly positive feedback to maintain the participant-therapist relationship or meet perceived expectations during the interview. While participants did mention some challenges and suggestions of improvement, additional negative experiences or less positive feedback might have emerged under different circumstances.

## Conclusions

In the present study, participants’ experiences of undergoing ICBT for climate change-related distress were investigated. The results suggest that participants found the treatment beneficial as it provided them with new tools for approaching and acting on their negative responses to climate change. Although most participants had positive comments about the treatment program, some also reported finding different aspects of it challenging. Altogether, these findings can inform future development of internet-delivered treatment for psychological symptoms associated with climate change. Given that evidence for effective and feasible interventions aiming to support people with climate change-related distress is just starting to emerge, further research in this area is sorely needed.

## Electronic supplementary material

Below is the link to the electronic supplementary material.

Supplementary Material 1

## Data Availability

The analysis is based on transcribed interview data which contain sensitive personal information. To maintain participants’ privacy and anonymity, data will not be shared openly.

## Abbreviations

ICBT: Internet-delivered Cognitive Behavioural Therapy
RCT: Randomized Controlled Trial
CBT: Cognitive Behavioural Therapy
CCI-15: ClimateCope Index
TA: Thematic Analysis
