# Supplementary material for: Experiences of undergoing internet-delivered cognitive behavioural therapy for climate change-related distress: a qualitative study
Source: BMC Psychiatry. 2024 Nov 6;24:775. doi: 10.1186/s12888-024-06212-1 (PMC11539731; doi:10.1186/s12888-024-06212-1)
Supplement: Supplementary file 1 — Supplementary Material 1 [file 12888_2024_6212_MOESM1_ESM.docx]

**Supplementary Material 1**

*Semi-Structured Interview Guide*

1. How did you experience undergoing treatment for climate change-related distress?
2. Which (if any) of the tools that were presented did you experience as helpful? In what way?
3. Have you utilized any of the tools after treatment termination? If so – how?
4. Has your eco-anxiety changed in any way as a result of treatment? If so – how?
5. Has your climate engagement changed in any way as a result of treatment? If so – how?
6. What improvements could be made to the treatment program?
7. Is there anything else that you would like to add regarding your experience of undergoing the ClimateCope treatment?
